# A Validation Protocol for an Instrumented Wheel: A Comparison with a Dual-Roller Handrim Wheelchair Ergometer

**DOI:** 10.3390/mps8020025

**Published:** 2025-03-03

**Authors:** Safiya Noury, Arnaud Hays, Nolwenn Poquerusse, Opale Vigié, Lorian Honnorat, Ilona Alberca, Mathieu Deves, Justin Regnaud, Arnaud Faupin

**Affiliations:** 1 Laboratoire J-AP2S, Université de Toulon, 83130 La Garde, France; 2 Laboratoire HIPE, Université Aix-Marseille, 13385 Marseille Cedex, France; arnaud.hays@univ-amu.fr (A.H.);

**Keywords:** parasport, wheelchair, power, propulsion, biomechanical

## Abstract

Measuring the propulsion performance of sport wheelchairs in ecological conditions remains complex due to variations inherent in real-world practice. This study aims to develop a validation protocol for an instrumented wheel designed to measure propulsion power under ecological conditions. The wheel’s precision was compared to that of the Lode Esseda roller ergometer, the gold standard for measuring the force exerted on both the left and right hands. Tests were conducted at three speeds (1, 2, and 3 m/s) on a multisport wheelchair. Results show a strong correlation between the two systems, confirmed by a repeated measures ANOVA test (*p* > 0.05) and a root mean square error (RMSE < 5%). Bland–Altman plots reveal good agreement despite discrepancies observed at high speeds, potentially due to mechanical limits. The proposed protocol validates the instrumented wheel and highlights the importance of multi-speed evaluation to ensure valid measurements in ecological conditions.

## 1. Introduction

With the upcoming Paris Paralympic Games, research in parasports has gained significant momentum. Wheelchair-based disciplines account for 13 of the 22 Paralympic sports, representing 86% of the events scheduled for the 2024 Paris Paralympic Games. One of the primary challenges is measuring forces and movements during wheelchair propulsion under ecological conditions—meaning field conditions that closely replicate actual wheelchair sport environments. This includes testing on various surfaces (e.g., hardwood courts or synthetic tracks), analyzing propulsion efficiency during accelerations and direction changes in team sports like wheelchair basketball and rugby, and assessing propulsion strategies in individual sports such as para badminton. These conditions allow for evaluating propulsion effectiveness, comparing different wheelchair models and settings, and studying how factors like speed, surface type, and fatigue impact performance. Understanding these mechanics is essential for optimizing equipment, preventing injuries, and ultimately improving athletic performance [1]. Numerous studies focus on systems that simulate wheelchair propulsion, such as dynamometers, ergometers, and treadmills [2,3,4,5]. These tools allow for measurements to be conducted in controlled laboratory settings. Additionally, instrumented wheels equipped with sensors for measuring propulsion forces in ecological practice environments provide an alternative to ergometers. Based on studies by Vegter et al., Limroongreungrat et al., and Van der Woude et al. [6,7,8], this solution has proven to be a promising avenue in this field.

Before utilizing these devices, it is critical to verify their accuracy and reproducibility by comparing them to gold-standard measurement tools. In this study, we chose to validate a new prototype of an instrumented wheel by comparing it to a Lode Esseda roller ergometer, an apparatus used to measure athlete performance in controlled settings. This approach ensures that the measurements from the instrumented wheel align with those of the ergometer, which serves as the reference tool [9]. While numerous studies emphasize the diversity of methods for validating measurement instruments in parasports [2,3,4,5,9,10,11,12], few focus on direct comparisons with reference devices such as ergometers [9]. Some studies have validated other types of propulsion force measurement devices, but these often lack standardized protocols or comparisons with reference tools [10,11,12].

The main objective is to validate the instrumented wheel by comparing its power output measurements to those of the Lode Esseda roller ergometer under standardized conditions for forward propulsion movements.

Furthermore, the validation results will offer insights into the accuracy of the instrumented wheel’s measurements and promote the use of this technology for evaluating parasport performance.

## 2. Measurement Tools

### 2.1. Lode Esseda Ergometer

The Esseda is a wheelchair ergometer developed and manufactured by Lode BV (Groningen, The Netherlands) in collaboration with the University of Groningen, the University Medical Center Groningen (UMCG), and the Center for Human Movement Sciences (Groningen, The Netherlands). The ergometer measures the wheelchair user’s force output using a load cell configuration and simulates wheelchair propulsion on the ground through an admittance-control feedback loop.

**Figure 1:**
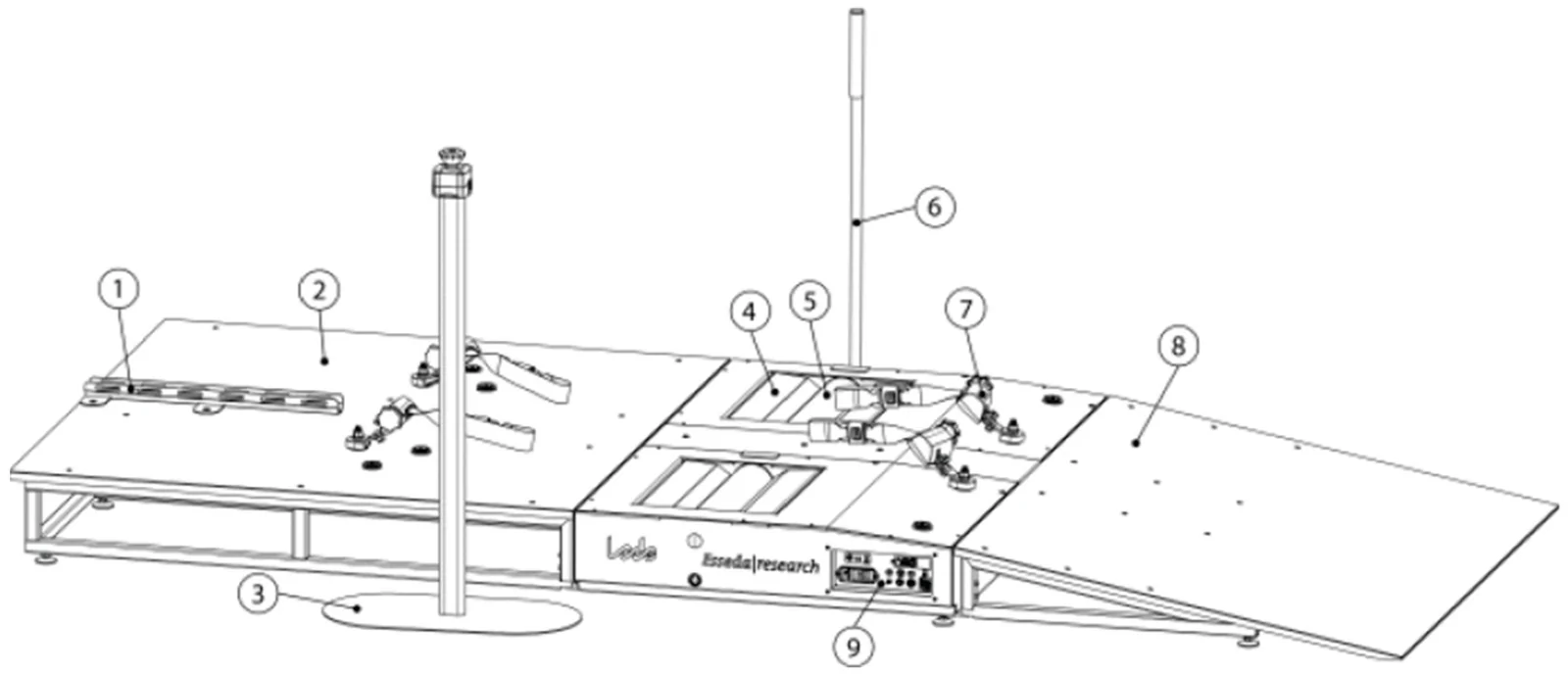
The Esseda wheelchair ergometer. Bottom: 1: wheeler extension; 2: castor support board; 3: emergency stop; 4: alignment flaps (4×); 5: roller (2×); 6: alignment handle; 7: straps (4×); 8: ramp; 9: communication module.

The Esseda ergometer is designed to replicate wheelchair propulsion under realistic conditions, functioning according to the principles of inertia. This device allows for resistance adjustments to simulate rolling conditions on various surfaces, effectively mimicking different levels of friction. The Lode Esseda ergometer is compatible with all types of wheelchairs, enabling athletes to use their personal equipment for testing. A flexible mounting system and an alignment tool ensure the correct positioning of the wheelchair relative to the ergometer’s rollers, providing an optimal setup for each user, regardless of the wheelchair’s size or specifications (Figure 1). Each roller is equipped with its own servomotor, control unit, and force sensor. The motor-to-roller gear ratio yields a maximum speed of 28 m/s. The sampling frequency for each wheel is 100 Hz. The ergometer is operated using the embedded closed-source software, developed and maintained by Lode BV in the C programming language. Its validation was conducted by De Klerk et al. in 2020 [9], who compared the Lode Esseda ergometer to an instrumented wheel at low velocities (1.1 m/s).

### 2.2. Instrumented Wheel: Mechanical Design

**Figure 2:**
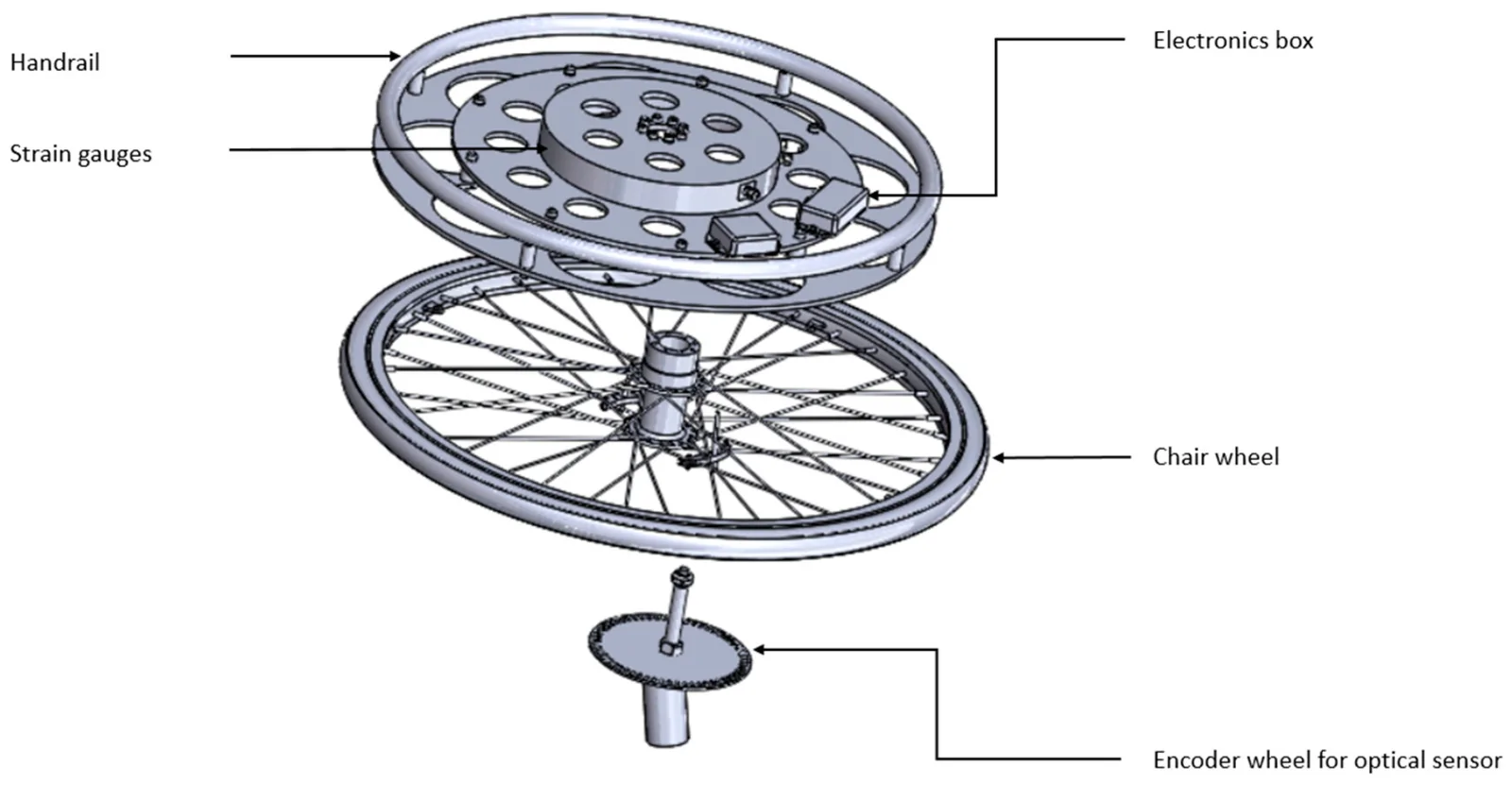
Exploded view of the instrumented wheel.

The instrumented wheel used in this study was specifically designed to measure the propulsion torque exerted by wheelchair athletes. This tool was developed in collaboration with the HIPE laboratory at Aix-Marseille University and the J-AP2S laboratory at the University of Toulon. The wheel is co-manufactured by Magtrol, a company based in Switzerland, and is equipped with two primary measurement systems:

- Optical Sensor: Positioned at the rear of the wheel hub, this sensor consists of a disk with 20 evenly spaced holes along a circular path, enabling rotation measurements at every 1/20th of a turn, or every 18°. This setup provides data on the angular position of the wheel during propulsion (Figure 2);
- Strain Gauges: Installed directly on the wheel, these gauges are designed to measure the forces applied by the athlete’s hand during propulsion, thereby quantifying the effort exerted directly on the wheel.

The instrumented wheel, constructed from an aluminum alloy, has a total weight of 5.2 kg. And, after recording the details of power consumption, the estimated battery life is as follows:

- 18 h in standby mode;
- 3.75 h in exercise mode.

These estimates provide an overview of the wheel’s operational duration under different usage conditions.

### 2.3. Instrumented Wheel: Software

In backup mode, the data stream is recorded directly on the wheel’s electronics at a sampling rate of 1000 Hz for torque data, along with cadence data. The cadence sampling frequency is determined by the wheel’s rotational speed; the faster the wheel rotates, the greater the number of sensor pulses, resulting in a more precise velocity measurement. The software, developed by the HIPE laboratory at the University of Marseille, generates text files that include all measured components for each wheel. Several metrics are captured by the software: torque, velocity, power, and ADC (analog-to-digital converter). The data directly measured by the wheel include ADC values and cadences (in rpm). Subsequently, the smartphone software calculates velocity, torque, and power.

To obtain more accurate power values, it is necessary to perform an offset using the OFFSET command in the software while the wheelchair is not under load. This adjustment is essential to ensure the validity of the torque measurement by determining the zero value. Once the offset value is acquired, the direction of rotation of the wheel can be set using the SENS mode, controlled via Bluetooth from the smartphone. The user must then roll forward for 2 s, which allows the sensor’s direction of rotation to be detected and recorded as the forward motion.

Subsequently, the conversion of ADC values to torque (N·m) and the measurement of cadences in rad/s allow for the calculation of power. Before converting to torque, it is necessary to subtract the recorded ADC offset value:

(1) ADC=Value−OFFSET   

The conversion of ADC values to millivolts (mV) is as follows:

(2) $${Value}_{mV}=\frac{ADC}{16}\;\;\;\;\;\;$$

This comes from the fact that the ADC resolution relative to the reference voltage (mV) is

(3) $$\frac{{Resolution}_{ADC}}{V_{ref}}=\frac{65.536}{4096}=16$$

Since the ADC is 16 bits, the resolution is $2^{16}=65.536$.

To convert this value to torque, it is necessary to convert it to microvolt (μV) and then divide it by the sensor’s sensivity, which is provided by the torque sensor manufacturer (MAGTROL), expressed in μV/N·m:

(4) $${Torque}_{Nm}=\frac{{Value}_{mv}\times 1000}{sensibility}=\frac{{Value}_{uV}}{sensibility}\;\;$$

where Value is the cadence value sent by the wheel every $\frac{1}{256}\;rpm$.

The sensitivity of the sensor is 33.400 μV/N·m. If the torque measurement is reversed, the final obtained value must also be inverted.

To convert cadence to rad/s, the value in rpm must be retrieved. Depending on the direction of rotation, it may be necessary to invert the data before converting to rad/s:

(5) $${Cadence}_{rpm}=Value\times 256\times sensRotation$$

(6) $${Cadence}_{rad/s}={Cadence}_{rpm}\times \frac{2\pi }{60}={Cadence}_{rpm}\times \frac{\pi }{30}\;$$

Finally, the determination of power is as follows:

(7) $${Power}_{W}={Torque}_{Nm}\times {Cadence}_{rad/s}\;\;\;\;\;\;$$

### 2.4. Protocol

**Figure 3:**
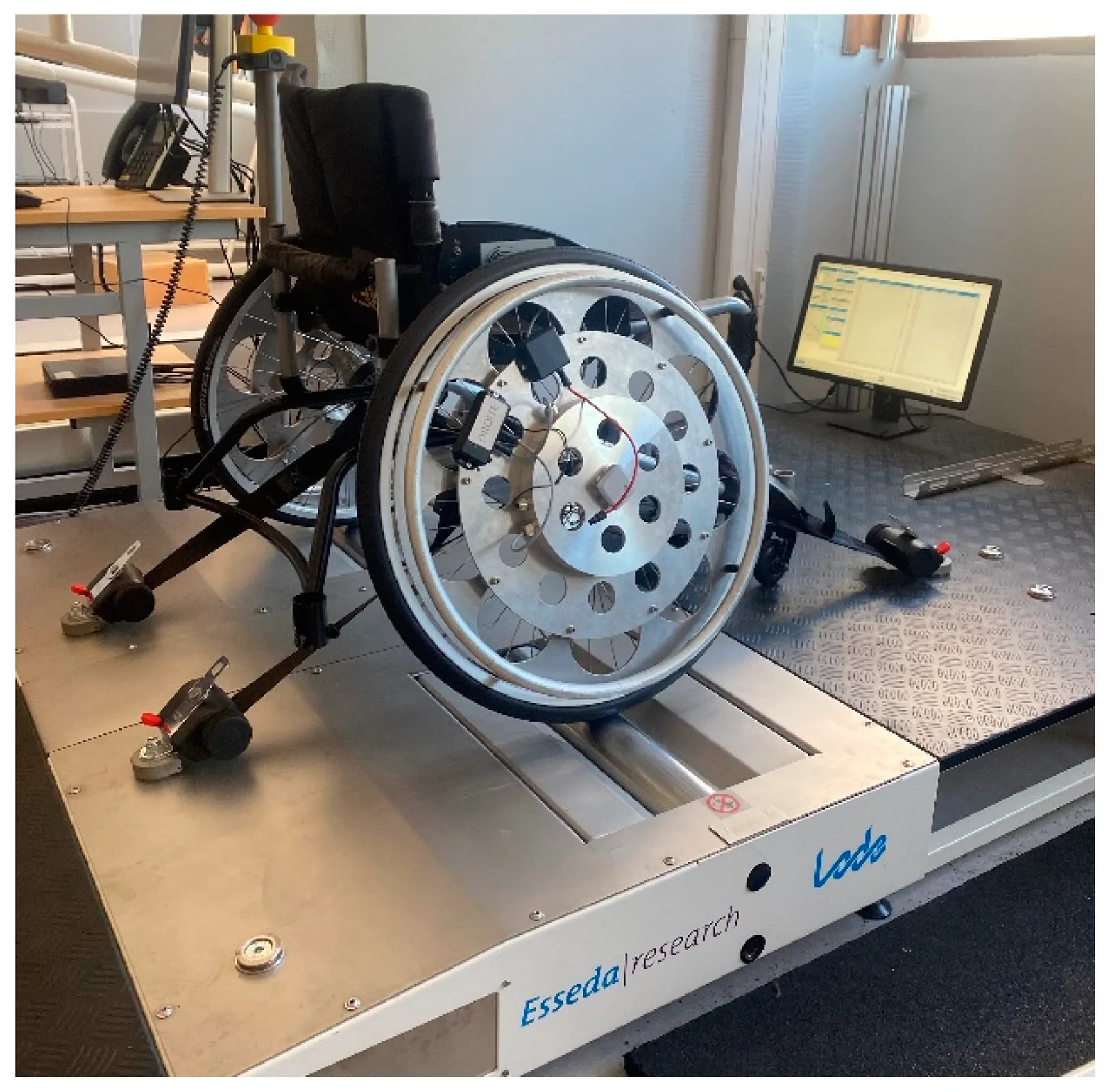
Mounting the wheelchair on the Lode Esseda ergometer.

This protocol aims to compare the Lode Esseda ergometer with the instrumented wheel at different velocities using a multisport wheelchair (MSW). To achieve this, an MSW equipped with instrumented wheels on both sides was mounted on the Lode Esseda roller ergometer. The wheel attachment remained consistent throughout all trials. The rollers of the ergometer drove the wheel at various rotational velocities (Figure 3):

Level 1: 1 m/s, equivalent to 3.6 km/h;
Level 2: 2 m/s, equivalent to 7.2 km/h;
Level 3: 3 m/s, equivalent to 10.8 km/h.

These correspond to low, moderate, and high propulsion velocities. Usma-Alvarez et al. (2011) recorded sprint velocities ranging between 2 m/s and 3 m/s, depending on the classification of rugby athletes using MSWs [13].

For each condition, a 15 s recording was conducted and repeated three times. A 5 min rest period was provided to the athlete between trials. The wheels were driven by the ergometer, and the athlete was instructed to perform pushes while remaining stationary. Synchronization was achieved using a velocity peak generated before stabilization, where the athlete applied an impulse to the wheels. The participant was a 27-year-old male weighing 70 kg, able-bodied, and practiced wheelchair sports once a week.

The Instrumented wheel was used to collect force data from the left and right hands on the handrim. The Lode Esseda ergometer served as the gold standard for measuring the force exerted on both the left and right hands.

### 2.5. Data Processing

Power data from the instrumented wheel and the Lode Esseda ergometer were exported as CSV files. The programming software used was Matlab R2023b (The MathWorks; Natick, MA, USA). To compare power data between the instrumented wheel and the Lode esseda ergometer, resampling by interpolation was conducted using Matlab’s interp1 function. Data from the instrumented wheel were resampled at 100 Hz. The data were then filtered to reduce the noise introduced by irregularities in the ergometer rollers and wheelchair tires. A 4th-order low-pass Butterworth filter with a cut-off frequency of 10 Hz was applied.

The ergometer served as the reference measurement tool as It has been previously validated as a standard for measuring forces on both the left and right hands [6]. Studies validating the roller ergometer have shown results comparable to those obtained with another validated instrumented wheel with a low measurement error [6,7,10].

### 2.6. Statistical Analysis

The analyses focus on power peaks, as they are common in sport performance studies to emphasize maximum values. Rhodes et al. [14] clearly demonstrated that speed peaks are closely linked to optimal performance in wheelchair rugby. Moreover, Janssen et al. [15] used power, force, and speed peaks as well as averages as performance indicators. In essence, mechanical power is the product of the applied force and movement velocity. Thus, speed peaks generally correspond to power peaks, as an increase in propulsion speed leads to a higher generated power. It is worth noting that power peaks were not averaged; each peak was recorded individually for each tested speed. Additionally, building on the work by de Klerk et al. [9], other validation parameters, such as push time, cycle time, and average power, were also considered.

Statistical analysis was performed to assess differences in mean measurements between the two tools. Initially, the Shapiro–Wilk test confirmed a normal distribution of all measured outcomes. Repeated measures analysis of variance (ANOVA) was conducted for each factor using the JASP software, version 0.18.3, to compare the effects of different factors. Mean values for these variables were calculated independently for each condition. The effect size was interpreted, according to Cohen (1988) [16], as small (η^2^ = 0.01), moderate (η^2^ = 0.06), and large (η^2^ = 0.14).

Post hoc tests were conducted following ANOVA when *p* < 0.05 (significance threshold). For each significant difference, Cohen’s effect size d was calculated as follows:

(8) $$\eta^{2}=\frac{mean(x_{0})+mean(x_{1})}{std(x_{0},x_{1})},$$

where $x_{0}$ is factor 1, $x_{1}$ is factor 2, and $std(x_{0},x_{1})$ is the standard deviation between them.

ANOVA allows for measuring the effect of two factors: the system (which compares the results of the ergometer to the Lode Esseda) and speed (which examines whether speed variations affect power values regardless of the system used). Additionally, the interaction between the system and speed provides a comparison of results for each system at a given speed.

To complement the statistical data, the root mean square error (RMSE) was calculated. RMSE quantifies the difference between “true” values measured by the Lode ergometer and the “experimental” values provided by the instrumented wheel. RMSE, a direct measure of prediction error, can be interpreted as an estimate of uncertainty by assessing how closely a model reflects observed reality.

This calculation method emphasizes larger errors due to the squaring of deviations [17,18].

To visualize the results, Bland–Altman plots were used. These graphical tools compare two measurement methods or instruments by evaluating the agreement as well as any systematic bias or random errors between two datasets. These plots display the difference between measurements on the y-axis and the mean of the two measurements on the x-axis. Two additional lines are drawn: the mean difference line, highlighting the average bias, and the agreement limits, set at ±1.96 times the standard deviation of the differences around this mean. These limits define an interval in which approximately 95% of discrepancies should lie, indicating the expected agreement level between the two methods if considered concordant.

## 3. Results

The results will be presented for only one wheel (the right wheel). The instrumented wheel is designed to function independently on either side of the wheelchair. Therefore, validating a single wheel is sufficient for establishing the validation protocol. Additionally, in the study by Limroongreungrat et al. [7], only the right wheel was used for system validation, further supporting our methodological choice.

**Figure 4:**
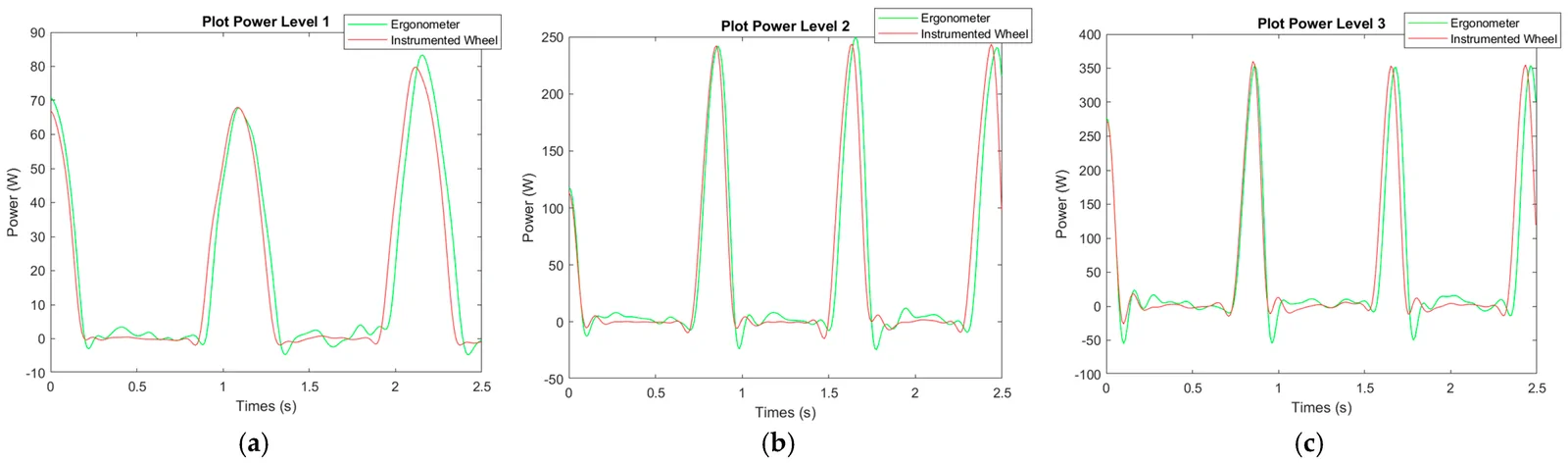
Comparison of power measurements between the ergometer and the instrumented wheel at level 1 (**a**), level 2 (**b**), and level 3 (**c**).

Below are the power graphs for the right wheel at different speed levels (Figure 4).

When comparing the graphs, the results from both measurement tools exhibit a similar trend and appear closely aligned, regardless of the ergometer’s speed.

### 3.1. ANOVA Test

The repeated measures analysis of variance (ANOVA) aims to determine whether the parameters under study (wheel speed and measurement system) have a significant effect on power measurement. The goal of the statistical test is to verify the null hypothesis, which states that the means of the two measurement systems are equal. Confirming this hypothesis would support the use of the instrumented wheel to measure forward propulsion power (Table 1).

The effect of speed tests whether, on average, speed has a significant impact on peak power, independent of the measurement system used. Significant results suggest that peak power varies depending on the tested speeds. There is high significance in the effect of speed in comparison to the ergometer (*p* < 0.001).

The system effect tests whether, on average, there is a significant difference in peak power measurements between the two systems, regardless of speed. If this test is not significant, it indicates that both systems measure similar peak power levels overall. In this case, for the right wheel, the system effect is not significant (*p* = 0.288). Additionally, the effect size is very small (η^2^ < 0.001), indicating an extremely limited influence of the measurement systems on peak power. Thus, while statistical differences may be detected, they are so minimal that they are unlikely to have practical implications.

Finally, the system–speed interaction tests whether the effect of the measurement system on peak power varies depending on speed. A significant result would indicate that one system measures peak power differently at various speeds. The results show that for both wheels, the difference between the two measurement systems is not significant (*p* = 0.608).

After the statistical tests, the root mean square error (RMSE) was calculated to assess the average difference between the values recorded by the instrumented wheel and those measured by the roller ergometer. Additionally, the results of the statistical tests for other parameters will be summarized for better readability. These parameters were processed using the same methodology described earlier.

### 3.2. RMSE

The results present the mean values of the parameters for each system studied, along with the differences between the two systems and the RMSE. Finally, the results of the ANOVA statistical test are provided for each parameter at each speed level. However, only the significance of the results is indicated, without displaying the exact values. The ANOVA results for other parameters (cycle time, push time, and average power) show no significant effect of the system or the system–speed interaction, while a significant effect of speed is observed. The RMSE obtained for the comparison of average power and peak power ranges from 0.06 W to 3.40 W, indicating a very low absolute error between the measured and predicted values**. These results reflect the good performance of the measurement system, which is the instrumented wheel, for the different observed parameters. Therefore, Table 2 reveals that the discrepancy between the two measurement tools remains below 5% for all studied parameters (RMSE < 5%). A clear trend is distinguished, where in the majority of cases, the data from the instrumented wheel are slightly higher than those recorded by the roller ergometer.

### 3.3. Bland–Altman Plots

**Figure 5:**
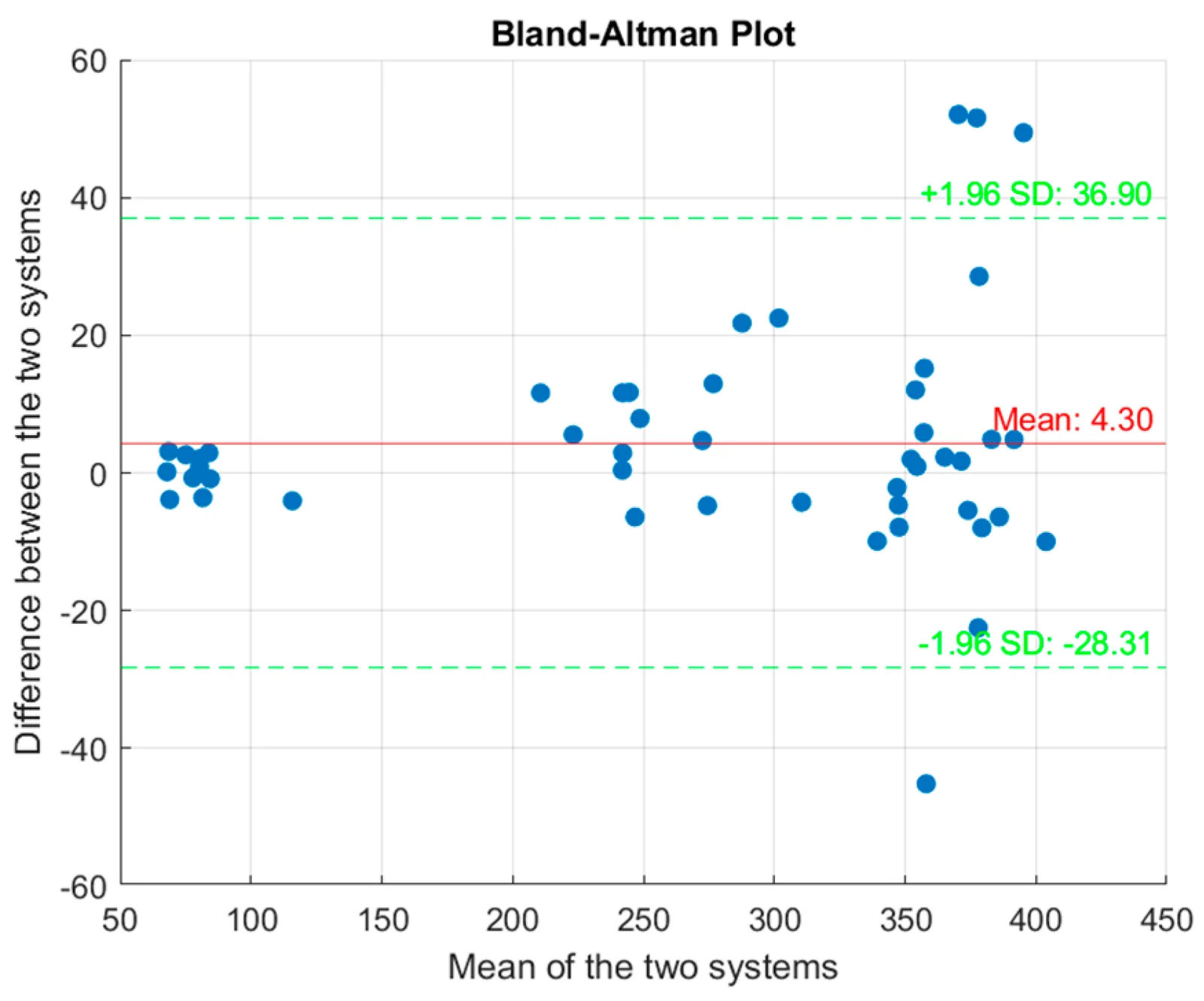
Bland–Altman plot.

We generated Bland–Altman plots for both right wheels, encompassing all tested velocity levels (Figure 5).

**Figure 6:**
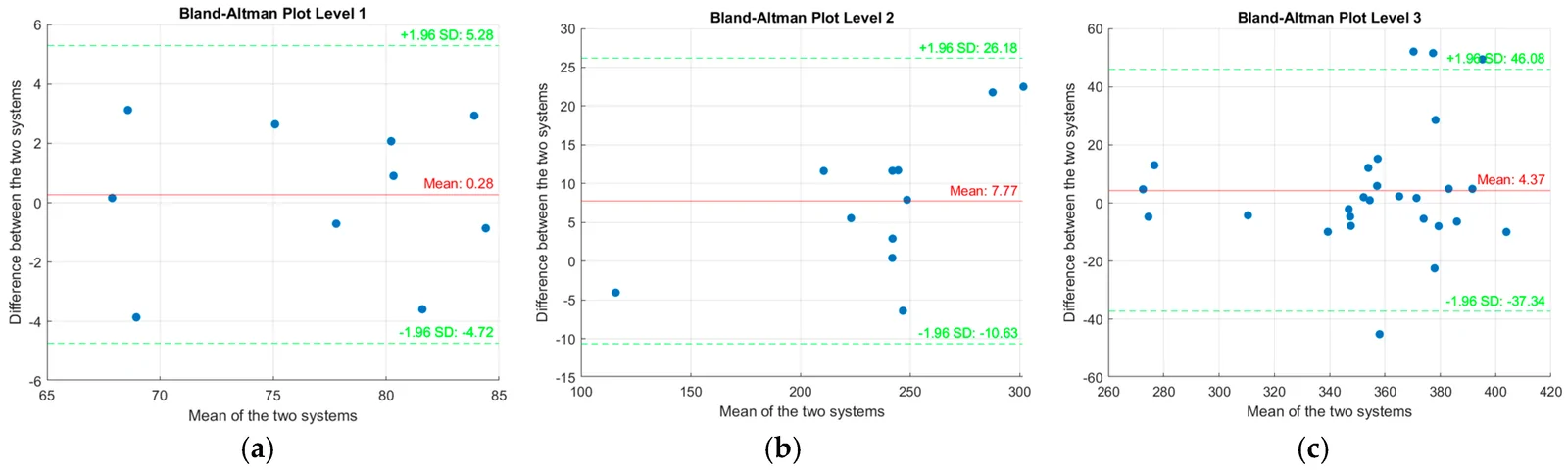
Bland–Altman plots for each velocity level for the right wheel (level 1: (**a**), level 2: (**b**), and level 3: (**c**)).

The majority of the data points lie within the agreement interval. However, for both wheels, four points fall outside this interval. To analyze the influence of velocity on agreement more precisely, a Bland–Altman plot was generated for each velocity level (Figure 6).

The results indicate that deviations from the concordance interval occur primarily at level 3, corresponding to the highest velocity. However, the majority of points remain within the concordance interval. Beyond analyzing the points outside the concordance interval, the average differences calculated for each velocity level reveal a systematic bias between the instrumented wheel and the ergometer. The mean differences are 0.28 at level 1, 7.77 at level 2, and 4.37 at level 3. These values demonstrate a relatively low average bias overall but show a notable increase at the intermediate velocity.

## 4. Discussion

This study aimed to develop a protocol for validating the instrumented wheel by comparing its power measurements to those obtained from the Lode Esseda roller ergometer during forward wheelchair propulsion in a controlled laboratory setting.

The results demonstrate a strong correlation between the peak power measurements obtained with the instrumented wheel and the roller ergometer (Figure 5). This correlation is supported by the absence of significant differences in the ANOVA results (Table 1 and Table 2), with only 0.1% of the total variance attributed to differences between the two systems. This indicates that both tools measure power and time similarly across all tested speeds.

Additionally, the estimates from the instrumented wheel show a deviation of less than 5% (Table 2) compared to values from the Lode ergometer. This confirms hypothesis 1, suggesting that the instrumented wheel provides valid and reproducible measurements relative to the Lode Esseda ergometer.

The Bland–Altman plots (Figure 5 and Figure 6) reveal variations in agreement between the instrumented wheel and the ergometer at different velocity levels, with more pronounced deviations at higher velocities. The four points outside the agreement interval occur exclusively at level 3, corresponding to the highest velocity. This may indicate that the wheel is more sensitive to high-speed conditions, potentially due to mechanical effects such as inertia, increased vibrations, or reduced wheel adherence to the ergometer roller, which could affect measurement accuracy. Indeed, according to the study by De Klerk [9] et al., the ergometer appears to slightly overestimate the generated force and torque. Furthermore, the study by Lancini et al. [19] highlights that rolling resistance and inertial parameters vary with speed, thereby influencing measurement accuracy. However, the majority of points fall within the agreement interval, indicating significant alignment between the two measurement tools. This suggests that these factors have minimal impact on the overall power measurement. Thus, the peak power values measured by the instrumented wheel are in agreement with those of the ergometer. Furthermore, it should be noted that the ergometer is only validated for a speed of 1.1 m/s. Consequently, the discrepancies observed at higher speeds could also be attributed to inaccuracies of the roller ergometer itself [9].

The analysis of differences also reveals a systematic bias between the instrumented wheel and the ergometer. The average bias is lower at level 1 (0.28) and increases at the high velocity (4.37), but it peaks at the intermediate velocity (7.77). This pattern may be explained by human factors, as the participant was not an expert in wheelchair propulsion and may have struggled to maintain consistent effort at intermediate speeds. In a sporting context, where velocity fluctuations are frequent. These findings highlight the need for a comprehensive validation protocol that not only tests agreement across all velocity levels but also aims to minimize bias at each level to ensure optimal measurement accuracy. However, the inclusion of zero within the agreement interval suggests that the observed bias may be due to data variability rather than a true systematic difference between the two tools, further supporting the validity of the results.

Future steps could involve developing a protocol to assess the precision of the instrumented wheel specifically for performance parameters under ecological conditions, such as the eight test, thereby allowing for the observation of directional changes frequently seen in wheelchair basketball and forward–backward propulsion movements frequently found in para badminton. These conditions aim to replicate field conditions. Planned improvements include reducing the wheel’s weight from 5.2 kg to closer to the 2 kg standard for competition wheels. Enhancements will also address precision, such as increasing the optical sensor’s resolution to 1/60th of a turn and integrating a gyroscope to refine velocity measurements, potentially resolving the bias observed at intermediate speeds.

Compared to other instrumented wheels on the market, notably the SMART wheel, which is frequently referenced in the literature [4,11], the wheel does not measure forces in three directions. The system focuses on measuring propulsion torque and angular velocity. This approach simplifies result interpretation and enables a direct calculation of the mechanical power generated by the athlete. Furthermore, our device is designed as a lighter and more portable alternative, allowing for installation on various sport wheelchair models without compromising maneuverability. It is important to note that this is only the first version of the instrumented wheel, and the second version will be tested using the same protocol.

## 5. Limit and Perspective

In this study, our primary objective was to establish a validation protocol for the instrumented wheel by comparing its power measurements with those of a reference roller ergometer, the Lode Esseda, in a controlled environment. For this initial validation phase, we deliberately chose a protocol involving a single able-bodied participant using a single wheelchair model. This methodological choice aimed to minimize inter-individual variability and focus on the metrological assessment of the device, ensuring that the observed discrepancies were attributable to the technical characteristics of the instrumentation rather than physiological or technical differences between subjects. However, the small sample size and controlled environment limit our ability to draw generalizable conclusions, particularly regarding the differences observed at speed level 3. Future studies should include a larger number of participants, including athletes with different pathologies, to assess the robustness and adaptability of the system under more diverse conditions. Once validated, this system will enable field tests to examine the impact of different surface types (wood, synthetic, track, etc.) on propulsion and exertion, analyze direction changes and accelerations in team sports such as wheelchair basketball and wheelchair rugby, evaluate propulsion efficiency to identify the most effective techniques, compare different wheelchair models or their settings (center of gravity position and wheel camber), measure the forces and torque applied to the wheels to better understand athletes’ propulsion strategies, and study propulsion variations based on speed, surface type, or fatigue. Thus, this instrumented wheel represents a significant advancement for performance enhancement in parasports by providing an innovative and precise tool for analyzing and optimizing wheelchair propulsion techniques. However, for future validation studies of this instrument or its improved versions, we recommend that these be conducted with both athletes with and without disabilities and in more ecological settings, such as on the track or in team sport situations.

## 6. Conclusions

This study confirmed the validity of the instrumented wheel for measuring propulsion power under controlled conditions. Moving forward, it would be beneficial to develop a validation protocol in conditions closer to real-world practice to assess the instrumented wheel’s effectiveness in field environments, where parameters are more variable. Such a protocol would enable the collection of valid field data validated by a methodology tailored to ecological conditions, thereby taking into account variables such as rolling surfaces or different wheelchair configurations. Additionally, planned improvements, such as reducing the wheel’s weight, increasing sensor precision, and integrating a gyroscope, will further enhance measurement accuracy. This knowledge could then be used to provide practical recommendations to athletes and their coaches, who are aiming to optimize their performance in wheelchair sports.

## Figures and Tables

**Table 1:** ANOVA test for peak power values between the instrumented wheel and the Lode ergometer. Significance: * (*p* < 0.05). ** (*p* < 0.01). *** (*p* < 0.001). NS: no significance (*p* > 0.05).

Parameters	p	$\eta^{2}$
Speed	<0.001 ***	0.941 NS
System	0.288 NS	<0.001 ***
Interaction effect	0.608 NS	<0.001 ***

**Table 2:** Comparison between the ergometer and instrumented wheel outcome parameters during propulsion.

Level	Variable	Mean Instrumented Wheel	Mean Ergometer Lode	Difference	RMSE	ANOVA		
						Level	System	Int
1	Peak power (W)	77.01	76.57	0.44	0.06	***	NS	NS
	Mean power (W)	19.31	19.54	−0.23	0.57	***	NS	NS
	Push time (s)	0.45	0.46	0.01	0.40	***	NS	NS
	Cycle time (s)	1.02	1.07	−0.05	0.21	***	NS	NS
2	Peak power (W)	238.72	248.46	−9.74	0.41	***	NS	NS
	Mean power (W)	40.87	39.89	0.98	1.31	***	NS	NS
	Push time (s)	0.27	0.25	0.02	0.38	***	NS	NS
	Cycle time (s)	0.78	0.79	−0.01	0.18	***	NS	NS
3	Peak power (W)	360.24	352.03	8.21	0.23	***	NS	NS
	Mean power (W)	44.04	42.59	1.45	3.40	***	NS	NS
	Push time (s)	0.20	0.19	0.01	0.22	***	NS	NS
	Cycle time (s)	0.76	0.77	0.01	0.16	***	NS	NS

Int: interaction effect. Significance: * (*p* < 0.05). ** (*p* < 0.01). *** (*p* < 0.001). NS: no significance (*p* > 0.05).

## Data Availability

The original contributions presented in this study are included in the article. Further inquiries can be directed to the corresponding authors.
